# Impact of Obesity on the IL-6 Immune Marker and Th17 Immune Cells in C57BL/6 Mice Models with Imiquimod-Induced Psoriasis

**DOI:** 10.3390/ijms24065592

**Published:** 2023-03-15

**Authors:** So Hee Park, Kyung Ah Lee, Jae-Hyeog Choi, SaeGwang Park, Dae-Wook Kim, So Young Jung

**Affiliations:** 1 Department of Dermatology, Haeundae Paik Hospital, College of Medicine, Inje University, Busan 48108, Republic of Korea; 2 Department of Plastic and Reconstructive Surgery, Haeundae Paik Hospital, College of Medicine, Inje University, Busan 48108, Republic of Korea; 3 New Drug Development Center, Daegu-Gyeongbuk Medical Innovation Foundation (K-MEDIhub), Daegu 41061, Republic of Korea; 4 Department of Microbiology and Immunology, College of Medicine, Inje University, Busan 47392, Republic of Korea; 5 Department of Orthopedic Surgery, Haeundae Paik Hospital, College of Medicine, Inje University, Busan 48108, Republic of Korea

**Keywords:** IL-6, imiquimod, obesity, psoriasis, Th17

## Abstract

Obese psoriatic patients experience higher disease severity and exhibit poorer treatment responses and clinical outcomes. It has been proposed that proinflammatory cytokines produced by adipose tissue exacerbate psoriasis; however, the role of obesity in psoriasis remains unclear. This study aimed to elucidate the role of obesity in the pathogenesis of psoriasis, focusing on immunological changes. To induce obesity, mice were fed a high-fat diet for 20 weeks. We then applied imiquimod to the skin on a mouse’s back for seven consecutive days to induce psoriasis and scored lesion severity every day for seven days. Cytokine levels in serum and the Th17 cell population in the spleen and draining lymph nodes were studied to identify immunological differences. The clinical severity was more remarkable, and histologically the epidermis was also significantly thicker in the obese group. Increased levels of IL-6 and TNF-α were observed in serum after psoriasis. They were elevated to a greater degree, with greater expansion of the functional Th17 cell population in the obese group. It is concluded that obesity could exacerbate psoriasis through mechanisms that involve elevated proinflammatory cytokine secretion and an expanded Th17 cell population.

## 1. Introduction

Psoriasis is a chronic inflammatory skin disorder that mostly manifests in the form of psoriasis vulgaris, characterized by raised, well-demarcated, erythematous plaques with silvery scales [1]. The mechanisms of pathogenesis for psoriasis remain poorly understood. In the past, hyper-proliferation and altered differentiation of keratinocytes was thought to be the main cause. It is now thought that complex alterations in keratinocytes, accompanied by immunological and vascular abnormalities, contribute to pathogenesis in psoriasis [2]. Among described immunological abnormalities, T-cell mediated processes, and the IL-23/Th17 axis in particular, have been implicated in psoriasis [3,4]. Th1-related cytokines such as IFN-γ and IL-12 were thought to be the driving force for pathophysiology in psoriasis. However, this understanding has evolved with the identification of IL-17-producing Th17 cells and their crucial role in psoriasis, which is analogous to their role in other autoimmune diseases, such as ankylosing spondylitis, multiple sclerosis, and Crohn’s disease [5]. IL-6, TGF-β, and IL-23 are required for Th17 cell differentiation. Initially, IL-6 and TGF-β drive naïve T-cell differentiation to Th17 cells, through activating STAT3 and subsequently promoting the transcription of Th17-specific genes such as *Rorc*, *Il17*, and *IL23r*. However, Th17 cells produced from TGF-β- and IL-6-driven-differentiation have limited pathogenicity and require subsequent exposure to IL-23 for their maturation and development into pathogenic Th17 cells. Finally, these pathogenic Th17 cells produce cytokines, including IL-17 and IL-22, which play crucial roles in psoriasis pathogenesis [6,7,8]. 

Psoriasis has recently been recognized as a form of systemic inflammation, and several clinical studies have indicated its linkage to psoriatic arthritis, cardiovascular disease, metabolic syndrome, and obesity [9]. Particularly, obese psoriatic patients show more severe clinical symptoms and poorer responses to regular treatment [10,11,12,13]. Additionally, weight loss alleviates symptoms of psoriasis [14]. Nevertheless, the role played by obesity in psoriasis is still unclear. 

Recent studies have indicated that elevated serum levels of IL-6 and IL-17 in mice with diet-induced obesity (DIO) promoted Th17 cell differentiation [15]. DIO was also associated with increased disease severity in Th17-dependent mouse models of disease, such as colitis and experimental autoimmune encephalomyelitis [15]. Accordingly, this study aimed to determine the potential role of obesity in exacerbating psoriasis in a C57BL/6 murine model with imiquimod-induced psoriasis by comparing the immunological differences between mice reared on obesity-inducing diet (OID) with mice reared on a regular diet (RD). In addition, this study is seeking a profound understanding of the role of obesity in the pathogenesis of psoriasis, based on immunological changes, helping determine appropriate immunomodulatory treatment in obese psoriatic patients.

## 2. Results

### 2.1. Severity of Skin Inflammation

**Figure 1:**
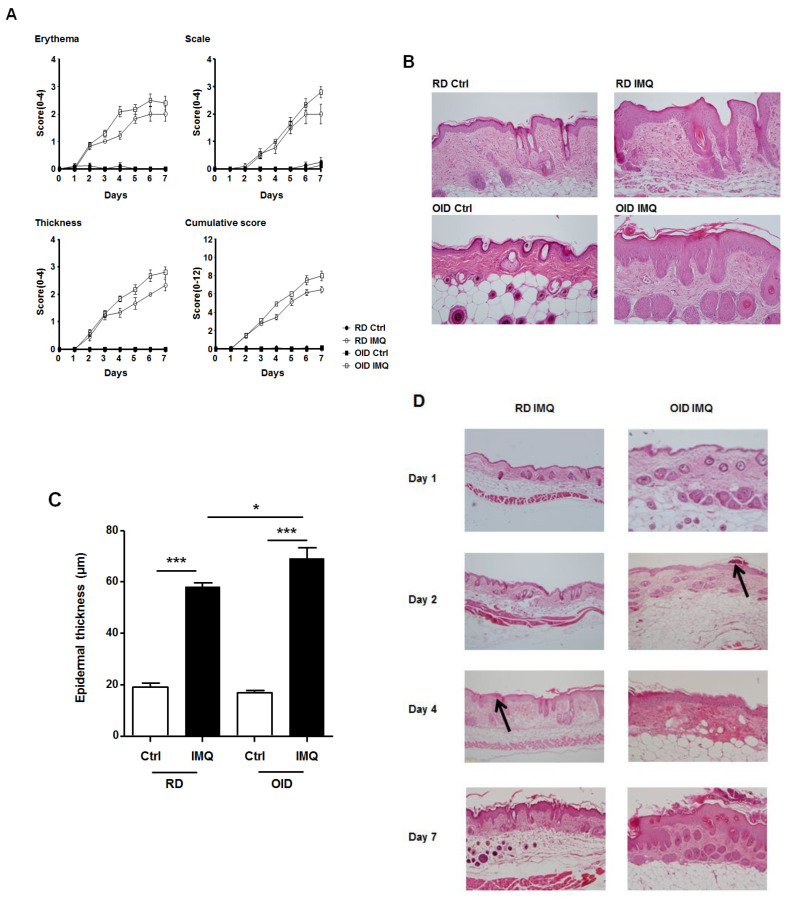
Exacerbated psoriasiform dermatitis and highly severe skin inflammation in imiquimod(IMQ)-treated mice reared on an obesity-inducing diet (OID) or regular diet (RD) (*n* = 4–6/group/each time point). Control (Ctrl) indicates the mice not treated with imiquimod. Subfigure (**A**) shows the scores of psoriasis symptom parameters within time while (**B**) depicts the epidermal thickness of RD and OID groups after seven days of imiquimod treatment. Skin tissue from the backs of mice was stained with hematoxylin and eosin, imaged at ×200 magnification. Subfigure (**C**) indicates the epidermal thickness, measured from the dermo-epidermal junction to the granular layer. Data are represented as means ± SEM. * *p* < 0.05, *** *p* < 0.001. Subfigure (**D**) depicts the histological changes in skin tissue samples from the backs of mice 1, 2, 4, and 7 days after imiquimod administration. Arrows in the third panel on the left and the second panel on the right indicate an intra-corneal microabscess, a characteristic feature of psoriasis. Imaged at ×100 magnification.

To assess the effect of obesity on the severity of skin inflammation, a topical imiquimod cream was applied to shaved skin on the backs of OID and RD mice for 7 consecutive days. Erythema and increases in skin thickness were observed from the second day, and scaling from the third day in the RD and OID groups. Scores on each symptom parameter increased up to the seventh day of the experiment and were significantly higher in the OID mouse group (Figure 1A). H&E staining revealed that the epidermis became thicker after 7 days of imiquimod treatment in the RD and OID groups (Figure 1B). However, the epidermal thickness was significantly greater in the OID group (69.2 ± 4.26 μm) than in the RD group (58.11 ± 1.52 μm) (Figure 1C). Histological changes were observed over the time course (Figure 1D). From the second day to the seventh day, there were visible changes in epidermal thickness and cell infiltration into the dermis. The epidermis gradually thickened with time and became prominent in the OID mice. Additionally, intra-corneal microabscess formation, a characteristic feature of psoriasis, was observed earlier in OID mice (at day 2) than in RD mice (at day 4).

### 2.2. Serum Levels of Psoriasis-Associated Proinflammatory Cytokines 

**Figure 2:**
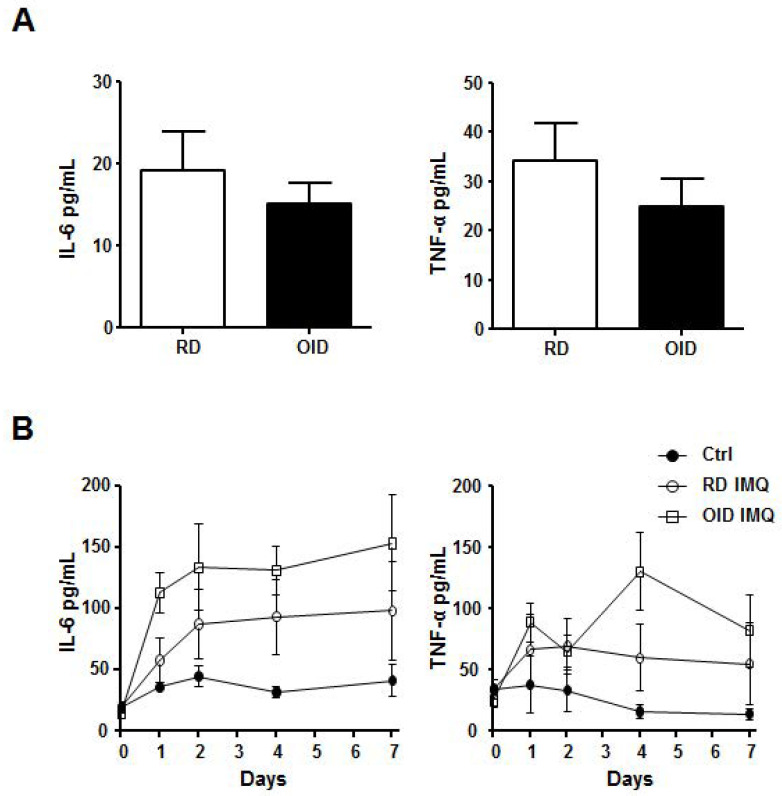
CBA-assayed proinflammatory cytokines IL-6 and TNF- α in imiquimod (IMQ)-treated mice reared on an obesity-inducing diet (OID), as compared to a regular diet (RD) (*n* = 4–6/group/each time point). Subfigure (**A**) represents the initial levels of cytokines measured prior to imiquimod treatment, while subfigure (**B**) presents them after 1, 2, 4, and 7 days from imiquimod treatment. Control (Ctrl) indicates the mice not treated with imiquimod. Data indicate the concentrations of cytokines ± SEM in serum and the results are representatives of two independently performed experiments.

We next examined if obesity affected the production of psoriasis-related proinflammatory cytokines IL-6 and TNF-α in the serum after imiquimod treatment. Serum samples were collected at 5 different time-points: before imiquimod treatment and days 1, 2, 4, and 7 after treatment. There was no difference in serum IL-6 and TNF-α between the OID and RD groups before imiquimod treatment (Figure 2A). IL-6 and TNFα increased one day after treatment, and elevated levels of both cytokines remained unchanged until the seventh day in both the OID and RD groups. Although the levels of the cytokines were higher in the OID group, there were no statistical differences from those in the RD group (Figure 2B).

### 2.3. Th17 Cell Population in Spleen and Lymph Node

**Figure 3:**
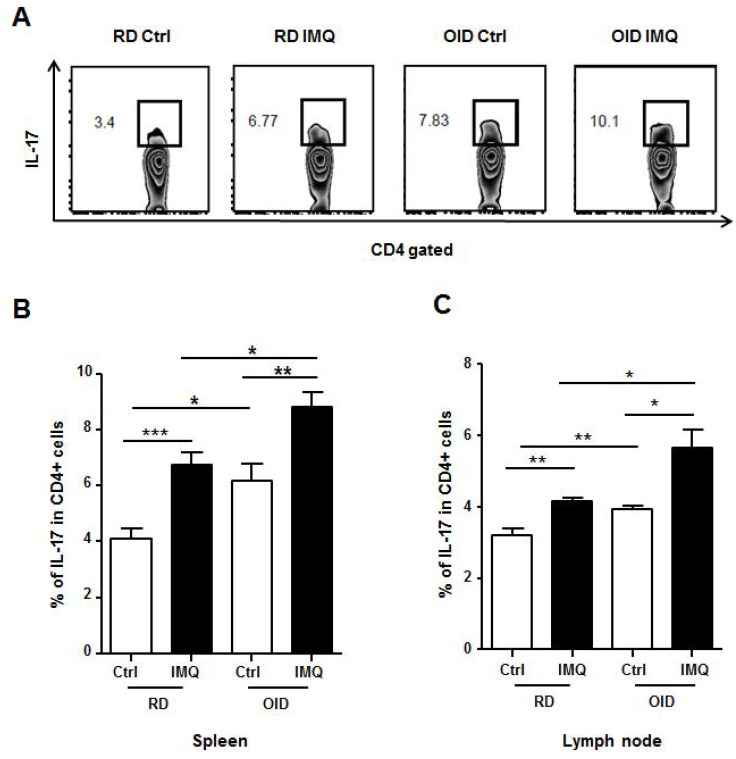
The population of IL-17-producing Th17 cells in the spleens and draining lymph nodes in imiquimod (IMQ)-treated mice reared on an obesity-inducing diet (OID) or regular diet (RD) (*n* = 4–6/group), seven days after imiquimod treatment. Single cells were isolated from spleens and lymph nodes, labeled with conjugated antibodies specific for CD3, CD4, and IL-17, and then analyzed by flow cytometry. (**A**) Gating used, (**B**) Spleen, (**C**) Lymph node. Data are represented as means ± SEM. * *p* < 0.05, ** *p* < 0.01, and *** *p* < 0.001. Results are representative of two independently performed experiments.

As Th17 cell populations have been identified to play a key role in the pathogenesis of psoriasis, we investigated cells obtained from spleens and lymph nodes at day 7 after imiquimod treatment. The strategy of IL-17^+^CD4 T cells gating was identified in Figure 3A. In the spleens, the highest percentage of Th17 cells was in the imiquimod-treated OID group (8.82 ± 0.51%), followed by the imiquimod-treated RD group (6.74 ± 0.45%), the non-treated OID group (6.15 ± 0.63%), and the non-treated RD group (4.10 ± 0.38%) (Figure 3B). As such, imiquimod treatment increased the percentage of Th17 cells in both the OID and RD groups. A more considerable increase in Th17 cells was observed in the imiquimod-treated OID group than in the imiquimod-treated RD group. The Th17 cell population was also larger in the non-treated OID group than in the non-treated RD group. The differences between the imiquimod-treated OID group and the imiquimod-treated RD group, and between the non-treated OID group and the non-treated RD group were statistically significant. In the draining lymph nodes, we observed similar trends in Th17 cells. The percentage of Th17 cells was the highest in the imiquimod-treated OID group (5.64 ± 0.51%), followed by the imiquimod-treated RD group (4.15 ± 0.09%) and the non-treated OID group (3.94 ± 0.11%), with the smallest percentage observed in the non-treated RD group (3.19 ± 0.19%) (Figure 3C).

### 2.4. IL-17 Secretion of Th17 Cell Population in Spleen and Lymph Node

**Figure 4:**
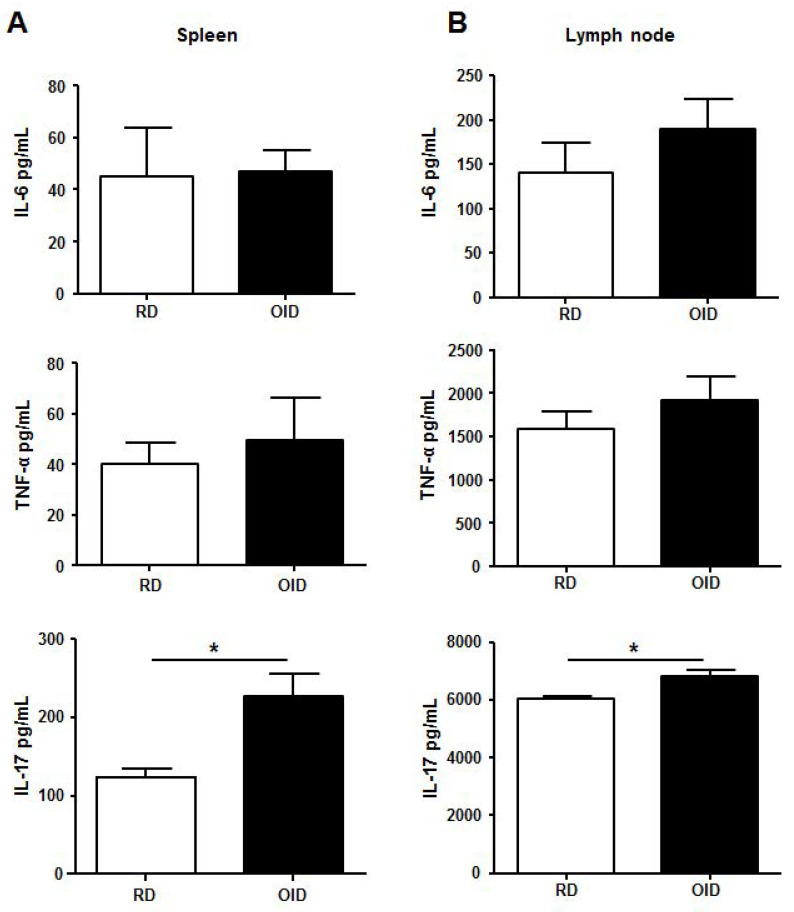
Level of cytokines (IL-17, IL-6, and TNF- α) secretions from spleen (**A**) and lymph node (**B**) in imiquimod-treated mice reared on obesity-inducing diet (OID) or regular diet (RD) (*n* = 4–6/group). The cytokines were measured in the cellular supernatants after stimulating the cells with anti-CD3 and anti-CD28 for 72 h. Data are represented as the mean cytokine level ± SEM. * *p* < 0.05. Results are representative of two independently performed experiments.

Th17 cells characteristically secrete IL-17. We previously found that the Th17 cell population was the largest in OID mice treated with imiquimod. IL-17 levels were measured to determine if increased Th17 cell counts led to increased cytokine secretion. Cells were isolated from the spleens and draining lymph nodes of mice in the imiquimod-treated groups and were stimulated in vitro with anti-CD3 and anti-CD28 for 72 h, following which cytokines in the culture supernatant were measured. The level of IL-17 was significantly higher in the OID group than in the RD group (Figure 4). Meanwhile, the levels of IL-6 and TNF-α were higher in the OID group than in the RD group, but the recorded differences between the two groups were insignificant.

## 3. Discussion

In this study, we investigated the role of obesity in psoriasis and how obesity aggravates psoriasis using an imiquimod-induced murine model of psoriasis in C57BL/6 mice. Imiquimod was observed to induce more severe psoriasis in obese mice, and the levels of IL-6, TNF- α, and Th17 cells, were simultaneously elevated.

Several different mouse models of psoriasis have been developed, including a genetically engineered model and models induced by xenotransplantation or transfer of immune cells [16]. In this study, we used the imiquimod-induced psoriasis mouse model, which was developed in 2009 by van der Fits et al. [17]. Imiquimod is a ligand for Toll-like receptor (TLR) 7 and TLR8 and is used in dermatology for the treatment of several cutaneous diseases, including actinic keratosis and basal cell carcinoma, as well as viral diseases such as condyloma accuminatum. It is known that the treatment effect of imiquimod in these diseases is mediated through activation of monocytes, macrophages, and plasmacytoid dendritic cells expressing TLR7 or TLR8 [18]. A recent study showed that TLR7 is also expressed in differentiated keratinocytes and that imiquimod can stimulate keratinocyte production of proinflammatory cytokines via NF-κB [19]. TLR7 and TLR8 are also expressed in the subcutaneous adipose tissue of the C57BL/6 mice [20]. Based on this information, we deduced that topical application of imiquimod could stimulate cytokine production via keratinocytes and immune cells as well as adipose tissue. For this reason, we chose an imiquimod-induced model of psoriasis for our experiments. 

Several clinical studies have associated psoriasis with obesity. Firstly, children who are overweight are at increased risk of early onset psoriasis [21]. The risk of developing psoriasis is higher in overweight or obese people, who also present with more severe clinical symptoms [22,23]. Treatment responses to both systemic and biological therapies are poorer in obese individuals [12,13,24]. Additionally, weight loss reduces the severity of psoriasis symptoms and increases response to systemic treatment [14]. We observed a similar relationship between obesity and severity of symptoms in an imiquimod-induced mouse model of psoriasis. Although OID and RD groups first showed similar clinical symptoms two days after imiquimod treatment, progression was more rapid in the first group, coupled with higher disease severity. Histological results showed that epidermal thickness was significantly greater in the OID mice. Additionally, collections of neutrophils in the stratum corneum (also known as Munro’s microabscess) which is a characteristic histologic finding in psoriasis tended to be seen earlier in the OID group than in the RD group. For histological examination, we made more than three slides stained with hematoxylin and eosin (H&E) with the skin obtained from one mouse. Munro’s microabscess, if present, was not always observed in all slides made from the same mouse. It is thought that whether Munro’s microabscess can be observed or not is dependent on where the tissue cut is sampled from. Additionally, there were biopsy results from the OID group, in which Munro’s microabscess was not found on the second day. However, it was found in the skin tissues of all the sacrificed mice on the fourth day. On the other hand, in the RD group, Munro’s microabscess was not found at all on the second day and began to be observed on the fourth day. Same as similar to the second day of the OID group, in the RD group, Munro’s microabscess was not observed in the skin tissue of all the sacrificed mice on all samples of the fourth day except one. Based on the results, we can predict that formation of Munro’s microabscess occurs earlier in the OID group. Taken together, the differences in clinical symptoms and histology suggest that in this disease model, OID exacerbates the progression of psoriasis, but does not appear to play a considerable role in initial psoriasis development.

Recently, obesity has come to be thought of as a condition of systemic inflammation. Chronic low-grade inflammation in obese individuals is associated with metabolic diseases, cancers, and psoriasis [25,26]. While the association between psoriasis and obesity is well known, the mechanism of this association and how it results in more severe pathophysiology remains unclear. It has been proposed that adipokines and proinflammatory cytokines produced by adipose tissue play a role in exacerbating psoriasis [9,27]. Accumulated adipose tissue in obese individuals do not simply act as an energy store, but also has endocrine and immune functions. Adipocytes and macrophages in adipose tissue can produce various proinflammatory cytokines, and elevated concentrations of IL-6 and TNF-α are increased in adipose tissue and serum during obesity [28,29,30]. It is thought that obesity-linked elevated cytokine levels are associated with accelerated macrophage accumulation in adipose tissue [31,32]. In this study, we found no differences in the serum levels of IL-6 and TNF-α in the OID and RD groups before imiquimod treatment. This is different from what previous studies have found [28]; however, we observed significantly higher IL-6 and TNF-α levels in the OID group than in the RD group after imiquimod treatment. Since TLR7 and TLR8 are expressed in the adipocytes of C57BL/6 mice [20], it can be explained that the enlarged adipose cells in the OID group were more stimulated by imiquimod, affecting more increased IL-6 or TNF-α secretion. It is unclear if the elevated IL-6 and TNF-α levels directly caused the increased disease severity in this model. However, both IL-6 and TNF-α are important immune mediators during initiation and maintenance periods for psoriatic lesions, and elevated cytokine levels would have either direct or indirect effects on disease severity in OID mice [33].

IL-6 has long been associated with the pathogenesis of psoriasis and is elevated in the serum and skin of patients with psoriasis [34,35]. IL-6 is known to be a cytokine that is essential for initial differentiation of Th17 cells, a key population of cells associated with pathogenesis in psoriasis [6,7,36]. A recent study showed that IL-6 was required for obesity-associated Th17 cell expansion during induction of EAE with myelin oligodendrocyte glycoprotein 35–55 [15]. We hypothesized in this study that elevated IL-6 would affect Th17 cell differentiation, and that increased numbers of Th17 cells would consequently result in more severe clinical symptoms in OID mice. To test this hypothesis, we analyzed Th17 cell populations present in the spleens and draining lymph nodes at day 7. Greater Th17 cell populations were observed in the RD and OID groups treated with imiquimod compared to their control groups. In addition, the imiquimod-treated OID group recorded the highest number of Th17 cells. As was previously reported [15], we found a larger Th17 cell population in OID mice than in RD mice without imiquimod treatment. We also tested the larger population of Th17 cells in the OID group treated with imiquimod for cytokine production. Cells were isolated from the spleens and draining lymph nodes, activated with anti-CD3 and anti-CD28 for 3 days, and elevated levels of IL-17 were observed. As mentioned before, the role of IL -23 is important for maturation of Th17 cells. Although we have not evaluated IL-23, we can predict that IL-23 would have worked well enough in our experiment. This is because IL-17 secretion was found to increase due to larger Th17 cell papulation in the spleens and lymph nodes of OID mice treated with imiquimod. However, our study is limited in its ability to explain fully the process of changing from non-pathogenic Th17 cells to pathogenic Th17 cells. In our further studies, to examine whether the Th17 cells are functional or not, we will investigate upstream cytokine IL-23 as well as downstream cytokine IL-17.

In summary, imiquimod more highly stimulated the production of IL-6 from enlarged adipose tissue, and this may have increased differentiation of IL-17-producing Th17 cells in OID mice than in normal ones. Higher levels of IL-17 produced by larger numbers of Th17 cells may be the reason for more severe psoriasis symptoms in OID mice than in RD mice.

## 4. Materials and Methods

### 4.1. Mice and Treatment

Female C57BL/6 mice aged 8–10 weeks were purchased from Orient Bio, Inc. (Sungnam, Republic of Korea). All mice were housed in the Animal Care Facility at the College of Medicine, Inje University. Mice were randomly divided into two groups and were fed either a control regular diet (RD group) or an obesity-inducing high-fat diet (OID group) for 20 weeks. The commercially available high-fat diet (Dyets, Inc. Bethlehem, PA, USA) consisted of 40% fat. The high-fat diet group were defined as having OID when the mean body weight reached > 3SD above the mean bodyweight of the regular diet group [28,37,38].

### 4.2. Induction of Psoriasis 

We used a method previously described by van der Fits et al. to induce psoriasis [17]. The experimental groups were as follows: RD control group, 24.63 ± 1.27 g (*n* = 36), RD disease group, 24.56 ± 1.09 g (*n* = 40), OID control group, 39.15 ± 4.27 g (*n* = 36), and OID disease group, 42.07 ± 6.38 g (*n* = 40). Briefly, mice were anesthetized with an intra-peritoneal injection of keratmin (100 mg/kg) and xylazine (10 mg/kg). Anesthesia was administered only on the first day for shaving. Mice had their backs shaved, and any remaining hair was completely removed with depilatory cream. A 2.5 × 3 cm rectangle was drawn on the back of all mice to demarcate the treated area. Mice in the induced psoriasis group were treated with commercially available 5% imiquimod cream (Aldara^®^; 3M Pharmaceuticals, Saint Paul, MN, USA). A daily dose of 80 mg of cream containing 4 mg of imiquimod was applied for 7 consecutive days. A vehicle cream, Vaseline, was similarly applied to the control treatment group. 

### 4.3. Physical Scoring of Skin Inflammation

Severity of psoriasis was measured every day for seven days using a method that was also developed by van der Fits et al. [17]. Two dermatologists performed the scoring evaluation to the groups in a blinded manner. The symptoms of erythema, scaling, and thickness were scored independently on a scale from 0–4: 0 representing no symptoms, 1 representing slight symptoms, 2 indicating moderate symptoms, 3 indicating marked symptoms, and 4 representing very marked symptoms. A cumulative score (scale: 0–12) was then calculated by adding the scores for all three symptoms.

### 4.4. Histopathology of Skin Tissue

Skin tissue from the backs of the mice was collected on days 1, 2, 4, and 7, and was fixed in 10% buffered formalin solution and embedded in paraffin. Sections (5 μm) were prepared and stained with hematoxylin and eosin (H&E). The tissue sections were histologically examined, and epidermal thickness was scored.

### 4.5. Serum Cytokine Levels

100 μL of blood was collected using a heparinized capillary tube from the retro-orbital vascular plexus at days 1, 2, 4 and 7. Additionally, the serum was separated and stored at −20 °C. Levels of IL-6, TNF-α, and IL-17 were measured in serum using a mouse Th1/Th2/Th17 cytokine Cytometrric Bead Array (CBA) kit (BD Biosciences, Franklin Lakes, NJ, USA).

### 4.6. Flow Cytometry

Single cells were prepared from the draining lymph nodes and spleens on day 7. Cells were incubated with an Fc receptor blocking antibody 2.4G2 for 5 min, following which they were stained with the following fluorophore-conjugated antibodies: CD3-APC and CD4-PE/Cy5. For intracellular IL-17 staining, 1.5 × 10^6^ cells/mL were stimulated with PMA (50 ng/mL) and ionomycin (750 ng/mL) for 6 h, and Golgi stop was added for the last 3 h, then labeled with anti-mouse IL-17 FITC antibody. Intracellular labeling was performed using the BD Cytofix/Cytoperm^TM^ kit (BD Biosciences) according to the manufacturer’s instructions. All antibodies were purchased from eBiosciences, Inc. (San Diego, CA, USA). FACS data were acquired using a FACS Canto II flow cytometer (BD Biosciences) and analyzed using FlowJo Software V10 (FlowJo LLC, Ashland, OR, USA).

### 4.7. T Cell Activation and Supernatants Cytokine Assay 

Single cell suspensions were prepared from spleens and draining lymph nodes from imiquimod-treated RD and OID mice on day 7. For T cell activation, single cells were plated at a density of 7.5 × 10^5^ cells/well (48-well culture plate) that had been precoated with anti-CD3 antibodies (0.5 μg/mL). Anti-mouse CD28 antibodies (1 μg/mL) were added to the cell cultures. After 3 days, cell-free supernatant was collected for cytokine detection by CBA kit (BD Biosciences, Franklin Lakes, NJ, USA).

### 4.8. Statistical Analysis

Statistical analysis was performed using GraphPad Prism (Version 6 for Windows; GraphPad Software, Inc., La Jolla, CA, USA). Data are represented as means ± SEM of at least two or three independently performed experiments. Differences between groups were compared using an unpaired t-test, and the level of significance was set at *p* < 0.05. *p* < 0.05, 0.01, and 0.001 are marked with *, **, and ***, respectively. 

## 5. Conclusions

In conclusion, this study demonstrated that obesity exacerbates psoriasiform dermatitis in an imiquimod-induced murine model of psoriasis. Clinical symptoms such as erythema, scales, and increased epidermal thickness were more severe, and a histological feature characteristic of psoriasis was observed earlier in obese mice. The aggravation of symptoms was related to increased production of the psoriasis-related proinflammatory cytokines IL-6 and TNF-α. Obesity resulted in increased differentiation to Th17 cells, and the greatest number of Th17 cells was observed with IL-17 production in obese mice with imiquimod-induced psoriasis. We propose that this model of imiquimod-induced psoriasis in obese mice might be valuable for investigation of the role that obesity plays in psoriasis and to test different modes of treatment for patients with psoriasis and obesity. 

## Data Availability

All the data are contained within the manuscript.
